# Reply to Stummer, W.; Thomas, C. Comment on “Hosmann et al. 5-ALA Fluorescence Is a Powerful Prognostic Marker during Surgery of Low-Grade Gliomas (WHO Grade II)—Experience at Two Specialized Centers. *Cancers* 2021, *13*, 2540”

**DOI:** 10.3390/cancers13225705

**Published:** 2021-11-15

**Authors:** Arthur Hosmann, Matthias Millesi, Lisa I. Wadiura, Barbara Kiesel, Petra A. Mercea, Mario Mischkulnig, Martin Borkovec, Julia Furtner, Thomas Roetzer, Stefan Wolfsberger, Joanna J. Phillips, Anna S. Berghoff, Shawn Hervey-Jumper, Mitchel S. Berger, Georg Widhalm

**Affiliations:** 1 Department of Neurosurgery, Medical University of Vienna, 1090 Vienna, Austria; arthur.hosmann@meduniwien.ac.at (A.H.); matthias.millesi@meduniwien.ac.at (M.M.); lisa.wadiura@meduniwien.ac.at (L.I.W.); barbara.kiesel@meduniwien.ac.at (B.K.); petra.mercea@meduniwien.ac.at (P.A.M.); mario.mischkulnig@meduniwien.ac.at (M.M.); martin.borkovec@skyforge.at (M.B.); stefan.wolfsberger@meduniwien.ac.at (S.W.); 2 Comprehensive Cancer Center—Central Nervous System Tumours Unit (CCC-CNS), Medical University of Vienna, 1090 Vienna, Austria; Thomas.roetzer@meduniwien.ac.at (T.R.); anna.berghoff@meduniwien.ac.at (A.S.B.); 3 Division of Neuroradiology and Musculoskeletal Radiology, Department of Biomedical Imaging and Image-Guided Therapy, Medical University of Vienna, 1090 Vienna, Austria; julia.furtner@meduniwien.ac.at; 4 Division of Neuropathology and Neurochemistry, Department of Neurology, Medical University of Vienna, 1090 Vienna, Austria; 5 Department of Pathology, University of California, San Francisco, CA 94143, USA; joanna.phillips@ucsf.edu; 6 Division of Oncology, Department of Medicine I, Medical University of Vienna, 1090 Vienna, Austria; 7 Department of Neurological Surgery, University of California, San Francisco, CA 94143, USA; Shawn.Hervey-Jumper@ucsf.edu (S.H.-J.); Mitchel.Berger@ucsf.edu (M.S.B.)

We greatly appreciate Dr. Stummer’s and Dr. Thomas’s interest in our study and their important comments [1]. Due to their pioneering work in the field of the application of 5-aminolevulinic acid (5-ALA) in brain tumor surgery [2,3,4], it is a special honor that we added “an important contribution to the field” according to the authors’ opinion.

We congratulate Jaber et al. [5] on conducting the first systematical single center study in a large patient cohort and again pioneering work in this field investigating the prognostic value of 5-ALA in World Health Organization (WHO) grade II gliomas. As stated in our manuscript [6] and the comment, we performed “the first study […] in two specialized independent centers”. As also stated in our manuscript, “We were able to confirm […] the findings of the recent study conducted by Jaber et al.” [5]. Therefore, the results of our current independent study conducted at two specialized centers confirm and thus highlight the importance of visible intraoperative 5-ALA fluorescence as a prognostic factor in pure WHO grade II gliomas as first shown in the single center study by Jaber et al. [5].

We also thank the authors for the effort of summarizing and comparing the results of both studies [5,6]. Indeed, the number of isocitrate dehydrogenase (IDH) wildtype low-grade gliomas (LGG) was lower in our cohort (*n* = 5) compared to the study of Jaber et al. [5]. However, the exact number of IDH wildtype LGG included in this study by Jaber et al. is not fully clear for us. According to Table 1 of your comment, 46 IDH mutated gliomas were included; i.e., 28 IDH wildtype gliomas should be expected in this cohort accordingly. Interestingly, according to the Results section, including Table 2 of the original publication of Jaber et al. [5], only 16 IDH wildtype gliomas were indicated. Nevertheless, a higher portion of IDH wildtype gliomas were analyzed in the study by Jaber et al. [5] compared to our study [6]. Due to the low number of IDH wildtype gliomas in our cohort (*n* = 5), the statistical power of a multivariate survival analysis was thus too low. Since we were not able to perform a multivariate analysis, we cannot compare our results based on univariate analysis with the findings of the multivariate analysis of Jaber et al. [5], which identified IDH mutational status and fluorescence as independent prognostic factors for survival. Ideally, future studies including multiple centers with a high number of IDH wildtype LGG would be desirable to finally clarify the precise relationship between 5-ALA fluorescence and molecular markers in LGG, such as IDH status.

We fully agree with the authors of the comment that visible intraoperative 5-ALA fluorescence is an unfavorable prognostic factor in pure LGG according to the data of both studies, and thus we also agree with the comment that early initiation of radio- and chemotherapy should be considered in these patients.
